# Assessing Diversity in the *Camelina* Genus Provides Insights into the Genome Structure of *Camelina sativa*

**DOI:** 10.1534/g3.119.400957

**Published:** 2020-02-11

**Authors:** Raju Chaudhary, Chu Shin Koh, Sateesh Kagale, Lily Tang, Siu Wah Wu, Zhenling Lv, Annaliese S. Mason, Andrew G. Sharpe, Axel Diederichsen, Isobel A. P. Parkin

**Affiliations:** * Agriculture and Agri-Food Canada, 107 Science Place, Saskatoon, SK, S7N0X2, Canada; † Department of Plant Sciences, University of Saskatchewan, 51 Campus Drive, Saskatoon, SK, S7N5A8, Canada; ‡ Global Institute for Food Security, 110 Gymnasium Place, Saskatoon, SK, S7N0W9, Canada; § National Research Council Canada, 110 Gymnasium Place, Saskatoon, SK, S7N0W9, Canada; ** Department of Plant Breeding, Justus Liebig University, Heinrich-Buff-Ring 26-32, 35392 Giessen, Germany; †† Plant Gene Resources Canada, 107 Science Place, Saskatoon, SK, S7N0X2, Canada

**Keywords:** Camelina, Domestication, cryptic species, Reference genome, Subgenome, related species

## Abstract

*Camelina sativa* (L.) Crantz an oilseed crop of the Brassicaceae family is gaining attention due to its potential as a source of high value oil for food, feed or fuel. The hexaploid domesticated *C. sativa* has limited genetic diversity, encouraging the exploration of related species for novel allelic variation for traits of interest. The current study utilized genotyping by sequencing to characterize 193 *Camelina* accessions belonging to seven different species collected primarily from the Ukrainian-Russian region and Eastern Europe. Population analyses among *Camelina* accessions with a 2n = 40 karyotype identified three subpopulations, two composed of domesticated *C. sativa* and one of *C. microcarpa* species. Winter type *Camelina* lines were identified as admixtures of *C. sativa* and *C. microcarpa*. Eighteen genotypes of related *C. microcarpa* unexpectedly shared only two subgenomes with *C. sativa*, suggesting a novel or cryptic sub-species of *C. microcarpa* with 19 haploid chromosomes. One *C. microcarpa* accession (2n = 26) was found to comprise the first two subgenomes of *C. sativa* suggesting a tetraploid structure. The defined chromosome series among *C. microcarpa* germplasm, including the newly designated *C. neglecta* diploid née *C. microcarpa*, suggested an evolutionary trajectory for the formation of the *C. sativa* hexaploid genome and re-defined the underlying subgenome structure of the reference genome.

*Camelina sativa* (L.) Crantz is an ancient oilseed of the Brassicaceae family that contributed to the human diet from the Bronze to the Middle Ages (Hjelmqvist 1979; Hovsepyan and Willcox 2008; Larsson 2013) before losing favor to higher yielding relatives. More recently it has shown potential to become a low-input high value oil crop for the food and feed industry (Faure and Tepfer 2016). Several advantages of this species have been reported (Brown *et al.* 2016; Ye *et al.* 2016) including the ability to yield well on dry and marginal lands and its unique seed quality traits (Gugel and Falk 2006), particularly its balanced omega fatty acids (Simopoulos 2002). However, improvements can be made to the crop such as increasing seed size for improved harvestability and reducing the glucosinolate content, which is an anti-nutritional in animal feed (Schuster and Friedt 1998; Amyot *et al.* 2018). Biologically, *Camelina* species have two crop habits, annual spring and biennial winter types (Berti *et al.* 2016). Most of the domesticated *C. sativa* are spring type, whereas the majority of its wild relatives are winter type. Genetic diversity is vital for developing a robust breeding strategy to identify and incorporate the necessary variation for further crop improvement. Thus far, different molecular approaches have been explored to study a range of *Camelina* germplasm including, RAPD (Vollmann *et al.* 2005), AFLP (Ghamkhar *et al.* 2010), SSR (Manca *et al.* 2013), and SNP marker analyses (Singh *et al.* 2015); all the studies concluded that there were low levels of genetic diversity available within spring type *C. sativa* compared to other oilseed crop species.

The genus *Camelina* has been reported in the literature to contain anywhere from 6 to 11 species, suggesting some taxonomic confusion (Warwick and Al-Shehbaz 2006; Brock *et al.* 2019). Latterly there appear to be between six and seven commonly accepted species belonging to the genus which range in chromosome number and ploidy level; namely *C. sativa* (2n= 6x = 40), *Camelina microcarpa* Andrz. ex DC. (2n = 12, 2n= 4x = 26, 2n = 6x = 40) (Martin *et al.* 2017), *Camelina hispida* (Boiss.) Hedge (2n = 2x = 14), *Camelina rumelica* Velen. (2n = 4x = 26), *Camelina neglecta* (2n = 2x = 12) (Brock *et al.* 2019) and *Camelina laxa* C.A. Mey. (2n = 2x= 12) (Galasso *et al.* 2015). The seventh species *Camelina alyssum* is more contentious since current accessions available within genebanks appear indistinguishable from and are inter-fertile with *C. sativa*; therefore, it was suggested that *C. alyssum* is a synonym of *C. sativa*, although this has yet to be adopted by genebanks (Martin *et al.* 2017; Al-Shehbaz 1987). Although there was a well-documented chromosome series for *C. microcarpa* until recently there were no reported sub-species; however, Brock *et al.* (2019) suggested that the smallest *C. microcarpa* karyotype (2n = 12) should be re-classified as a new species, *Camelina neglecta*. Currently cultivated *C. sativa* is considered to be hexaploid with 20 chromosomes in a haploid set, while at least one of the related species (*e.g.*, *C. microcarpa*) has the same chromosome number (Francis and Warwick 2009) most have lower numbers. The genome sequence of *C. sativa* suggested a neopolyploid that had evolved from three lower chromosome number species, specifically one n = 6 and two n = 7 species (Kagale *et al.* 2014). *Camelina* species such as *C. neglecta*, *C. laxa* and *C. hispida* possess the same haploid chromosome numbers as subgenomes of the hexaploid and recent work has proposed that *C. neglecta* and *C. hispida* could indeed be extant progenitors of *C. sativa* (Mandáková *et al.* 2019). The study of these lower ploidy species could be instrumental in defining the relationship among the species as well as uncovering the polyploidization history of *Camelina* (Brock *et al.* 2019). Defining the relationships between these species at the subgenome level may also help to identify those species that are potential novel sources of allelic variation for introgression into *C. sativa*.

*Camelina microcarpa* has been of interest in studies of *Camelina* diversity as it is believed to be the closest extant relative to domesticated *C. sativa* and could help in understanding the domestication process in *Camelina* species, as well as providing novel variation (Brock *et al.* 2018). The collections of *C. microcarpa* species in different genebanks suggest that it has a diverse range of origin including the Mediterranean region, Armenia (Brock *et al.* 2018), Germany, Poland, Czechia, Slovakia and Georgia (Martin *et al.* 2017; Smejkal 1971). Diversity studies, analyses of genome size and chromosome number along with the success of hybridization efforts between *C. microcarpa* and *C. sativa* (Séguin-Swartz *et al.* 2013; Martin *et al.* 2019) suggested the close relationship between these two species (Brock *et al.* 2018; Martin *et al.* 2017). However, not all the results were so encouraging with varying levels of hybridization success depending on the genotype (Séguin-Swartz *et al.* 2013). These results were likely due to confusion with the classification of *C. microcarpa* accessions, either due to disparities in chromosome number and/or crosses being attempted with completely different species such as *C. neglecta* (Brock *et al.* 2019; Martin *et al.* 2017). Such anomalies could have led to an assumption of higher diversity within *C. microcarpa* species, with the discovery of *C. neglecta* in particular there is a need to better understand the relationship between the different accessions of *C. microcarpa* and *C. sativa* for potential utilization of such germplasm in *Camelina* breeding programs.

Estimation of genome size using flow cytometry and chromosome counts are common tools to infer ploidy in a species (Johnston *et al.* 2005; Martin *et al.* 2017; Brock *et al.* 2018; Séguin-Swartz *et al.* 2013). Complementary genomic tools can assist in clearly defining evolutionary relationships between species and in the case of *Camelina*, the available reference genome for *C. sativa* can facilitate such analyses (Kagale *et al.* 2014). Here, we explored genetic diversity using predominantly genotyping by sequencing (GBS) in different *Camelina* species, with a focus on *C. microcarpa*. The analyses of these related species suggested a group of *C. microcarpa* lines could represent a novel cryptic species. In addition, the subgenome structure of the *C. sativa* reference genome was re-defined and will provide a basis for utilization of the related species in *C. sativa* breeding. For example, this study identified a range of potentially valuable minor alleles from *C. microcarpa*, including those in three flowering related genes which may have impacted the *Camelina* domestication process.

## Materials and methods

### Plant materials

This study included a collection of 160 *C. sativa*, 27 *C. microcarpa*, two *C. alyssum*, one *C. neglecta*, one *C. laxa*, one *C. hispida and* two *C. rumelica* to establish the genetic relationship among the accessions (Table S1). The accessions were mainly obtained from Plant Genetic Resources of Canada in Saskatoon (http://pgrc3.agr.gc.ca/). One accession, “Midas”, was a commercial Canadian variety and 12 accessions were commercial varieties from the United States and Europe. Five accessions are breeding lines from the Agriculture and Agri-Food Canada Saskatoon Research and Development Centre (provided by Dr. Christina Eynck) and the remainder of the lines were thought to originate from eastern Europe and the Russian-Ukraine region and were donated from the National Centre for Plant Genetic Resources of Ukraine in Kharkiv.

### Flow cytometry analysis

The relative genome sizes of six different *Camelina* species were measured using flow cytometry according to the method described in Garcia *et al.* (2004). Approximately 1 cm^2^ of leaf tissue of both sample and an internal standard was placed in a plastic petri dish with 2 ml of Galbraith buffer (Galbraith *et al.* 1983), the mixture was chopped up with a razor blade and the solution was supplemented with 200 µg of ribonuclease A, before being filtered through a filter with a pore size of 30 µm. Propidium iodide was then added at a concentration of 60 µg/ml. The stained solution was kept at 4° for 2 hr and allowed to incubate at room temperature for an hour before taking measurements. DNA content of the nuclei from each species was estimated using fluorescence measurements with a green laser (532 nm) in a CyFlow Space Flow Cytometer (Partec). *Camelina sativa* (TMP23992) having known ploidy level and genome size (Kagale *et al.* 2014; Martin *et al.* 2017) was used as an internal standard to estimate the genome size of lower ploidy species. For all accessions three biological replicates were used.

### Chromosome counts

For this study, seeds from six accessions (*C. sativa* TMP23992, *C. neglecta* PI650135, *C. hispida* PI650133, *C. microcarpa* CN119243, *C. microcarpa* TMP24026 and *C. microcarpa* TMP23999) were germinated on moist filter paper in Petri dishes at room temperature. Chromosome counts were carried out based on the protocols detailed in Harrison and Heslop-Harrison (1995) and Snowdon *et al.* (1997) with minor modifications. Growing root tips (1-2 cm) were collected into tubes containing 0.04% 8-hydroxyquinoline solution (290 mg 8-hydroxyquinoline powder dissolved in 1 L H_2_O via treatment at 60° for 2 hr, then stored at -4° until use). The root-tip-containing solution was incubated in the dark for 2 hr at room temperature followed by incubation at 4° for 2 hr. Cells were fixed with Carnoy’s I solution (3 parts ethanol to 1 part glacial acetic acid) for 2 days at room temperature. After fixation the root tips were stored in 70% ethanol at -20°. The fixed root tips were rinsed twice for 10 min with distilled water to remove the fixative and incubated in 0.1 M pH 4.5 citrate solution (1.47 g trisodium citrate-dihydrate (Na_3_C_6_H_5_O_7_0.2H_2_O) and 1.05 g citric acid monohydrate (C_6_H_8_O_7_.H_2_O) in 500 mL water) for 15 min at room temperature followed by incubation in enzyme solution (0.25 g (5%) Onozuka R-10 cellulase and 0.05 g (1%) pectinase in 5 mL citrate solution) for another 30-40 min at 37°. Root tips were washed with distilled water for 30 min and placed onto a slide with a few drops of Carnoy’s I solution. On the slide, the root tissue was scrambled with a pin and left until the solution dried. Finally, a drop of DAPI staining solution VECTASHIELD Antifade Mounting Medium with DAPI (4,6-diamidino-2-phenylindole; product number H-1200 from Vector Laboratories) was added and covered with a coverslip before observing under UV fluorescence using a Leica DRME microscope at 1000 × magnification.

### DNA extraction

Immature leaf samples were collected for DNA extraction. Leaf tissue was stored at -80° prior to DNA extraction. All the samples were freeze-dried for at least 48 hr before lysis. DNA extractions were performed using a CTAB method (2% CTAB, 100mM Tris-HCl, 20mM EDTA, 1.4M NaCl) (Murray and Thompson 1980). After DNA extraction, samples were treated with RNase at 37° to remove RNA contamination. Quantification of DNA was performed with Quant-iT PicoGreen dsDNA Assay Kit (ThermoFisher Scientific) through fluorescence measured (485nm/535nm, 0.1s) using the Victor *X*Plate Reader (PerkinElmer).

### Library preparation and DNA sequencing

Genotyping was performed by an established GBS method (Poland *et al.* 2012). After DNA normalization (20 ng/ul), 200 ng of DNA were digested with *PstI* and *MspI* at 37° for 2 hr. Next, adapters were ligated to the restriction digested DNA fragments using T4 DNA ligase at 22° for 2 hr. The products were inactivated before multiplexing and 96 samples were pooled into a single library. After pooling, the library was amplified with a short extension time (30 sec) and purified using a QIAquick PCR Purification Kit (Qiagen). The final libraries were quantified using a Bioanalyzer (Agilent Technologies) to confirm the fragment size and quality of the library. Sequencing of 35 *C. sativa*, 9 *C. microcarpa*, 1 *C. rumelica* and one *C. alyssum* were completed on an Illumina HiScan SQ module (paired-end 100 bp reads) and the remainder were sequenced on an Illumina HiSeq2500 platform (paired-end 125 bp reads).

### DNA sequence analysis

An existing pipeline was used to demultiplex the reads and trim the reads for adapters, short reads and poor quality data using Trimmomatic (Bolger *et al.* 2014). Leading and trailing bases with quality below 15 and reads shorter than 55 bp were removed prior to mapping to the reference genome. The trimmed sequence reads were aligned with the reference genome of hexaploid *C. sativa* (Kagale *et al.* 2014) using Bowtie2 (Langmead and Salzberg 2012). In bowtie2 mapping, *–local* with *-sensitive* parameters were used with –score-min of L,0,0.8. In addition, a custom perl script was used to extract the single best unique hits. Obtained binary files (BAM) were used for variant calling as well as mapping sequence distribution. BEDTools (Quinlan and Hall 2010) was used to extract mapped reads and calculate the frequency of mapped reads along 100 Kb bins in the genome. Circos (Krzywinski *et al.* 2009) was used to plot the distribution of mapped reads along the *C. sativa* reference genome for the diploid, tetraploid and hexaploid *Camelina* genotypes. *UnifiedGenotyper* with standard parameters from the Genome Analysis Toolkit (McKenna *et al.* 2010) was used to call SNPs.

### Population differentiation

Obtained SNPs were analyzed for average dissimilarity between genotypes and Principle Coordinate Analysis (PCoA) was performed utilizing AveDissR Package (Yang and Fu 2017) in the R program (R Core Team 2017). Population structure was determined using Bayesian technique in STRUCTURE (Pritchard *et al.* 2000) with a burn-in period of 150,000 steps and 150,000 MCMC replicates where parallelization was performed with StrAuto tool (Chhatre and Emerson 2017). To determine optimal K, three replications were run with each value of K from 1 to 10. The value of K was converted into LnP(K) to obtain the plateau of ΔK. The optimal K was determined using the online version of “Structure harvester” (Earl 2012). PowerMarker (Liu and Muse 2005) was used to calculate gene diversity, Polymorphic Information Content (PIC) and Nei’s (1983) based genetic distance between the genotypes. MEGA 7 (Kumar *et al.* 2016) was used to construct the Neighbor Joining (NJ) tree among the genotypes. The phylogenetic tree was confirmed through the use of the maximum likelihood method (Tamura and Nei 1993) in MEGA 7 using bootstrap consensus tree (Felsenstein 1985) inferred from 1000 replicates, no significant differences were noted between the alternate tree structures (Figure S5). Analysis of Molecular Variance (AMOVA) and pairwise F_ST_ were calculated using GeneAlEx 6.5 (Peakall and Smouse 2006, 2012).

### Subgenome dominance

Data previously published by Kagale *et al.* (2016) was re-analyzed. The expression data from 12 tissues of *C. sativa* were arranged according to the re-defined subgenome structure and filtered for expression less than 0.01 TPM for all replicates. The 12 tissues were Germinating Seed (GS), Cotyledon (C), Young leaf (YL), Root (R), Stem (S), Senescing leaf (SL), Bud (BUD), Flower (F), Early seed development (ESD), Early mid seed development (EMSD), Late mid seed development (LMSD) and Late seed development (LSD). Filtering provided data for a range of expressed triplicated genes, from 9149 in LSD to 12634 triplets in Root (Table S10), which were analyzed for subgenome dominance in *C. sativa*. The analysis was performed using analysis of variance techniques where effects due to replication were kept as random. Genes that were expressed significantly (*P-value* <0.05) higher in any subgenome compared to the other two were considered dominant.

### Data availability

Supplemental data (Tables S1-S10; Figures S1-S6), as well as variant data, are provided through figshare: https://doi.org/10.25387/g3.11299280.

## Results

### Identification of ploidy series among Camelina species

**Figure 1:**
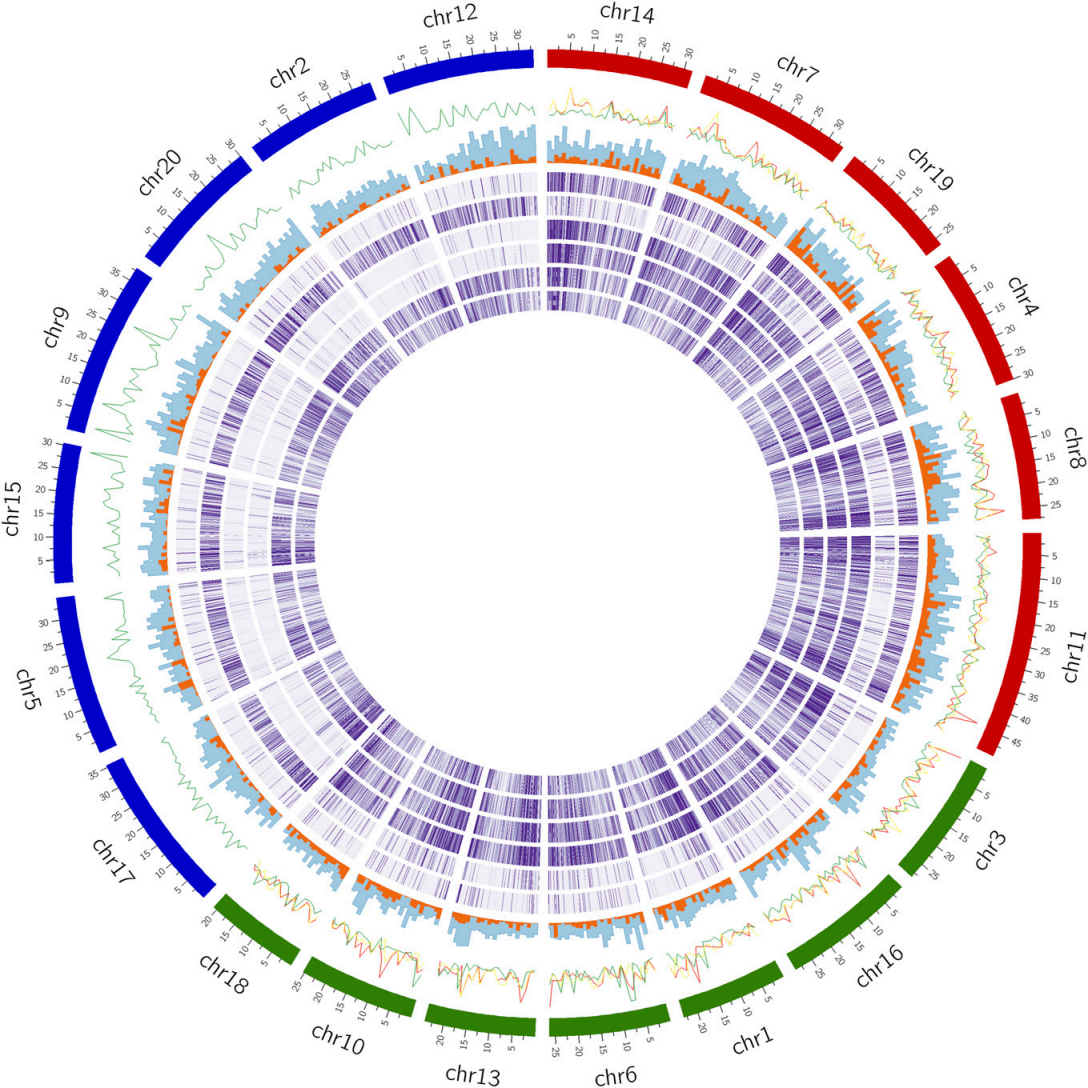
Identification of ploidy in *Camelina* species using genotyping by sequencing (GBS) data. From outer to inner track: 1) Clockwise three subgenomes of *C. sativa* reference genome in red, green and blue; 2) F_ST_ distribution across the genome: *C. sativa* *vs.* *C. microcarpa* “Type 1” in green, *C. sativa* *vs.* *C. microcarpa* “Type 2” in red and *C. microcarpa* “Type 1” *vs.* *C. microcarpa* “Type 2” in yellow; 3) SNP distribution of *Camelina* species in 1 Mb bins in blue and filtered SNPs in orange; 4-9) Heat maps showing read alignment of diploid genotype *C. neglecta* (PI650135), *C. hispida* (PI650133), tetraploid *C. microcarpa* (CN119243), *C. microcarpa* “Type 2” (TMP23999), *C. microcarpa* “Type 1” (TMP26172) and *C. sativa* (TMP23992) to the reference genome.

**Figure 2:**
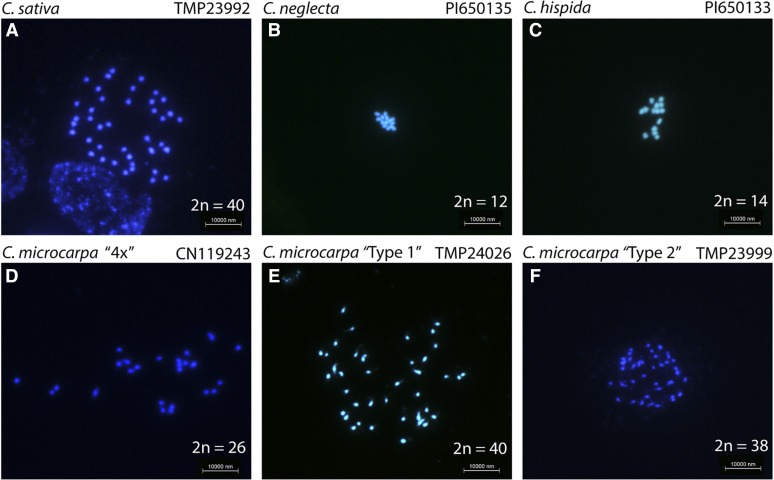
Chromosome counts for different *Camelina* species. a) *C. sativa* TMP23992 (2n = 40); b) *C. neglecta* PI650135 (2n = 12); c) *C. hispida* PI650133 (2n = 14); d) *C. microcarpa* “4x” CN119243 (2n = 26); e) *Camelina microcarpa* “Type 1” TMP24026 (2n = 40); and f) *C. microcarpa* “Type 2” TMP23999 (2n = 38).

GBS was performed for 193 *Camelina* accessions, high-quality sequence reads were aligned to the reference genome of *C. sativa*, DH55 (Kagale *et al.* 2014). The number of reads per line and alignment rate is summarized in Table S2. As expected, consistent read coverage was found across all 20 linkage groups of the reference genome for all accessions of *C. sativa* and *C. alyssum*. However, for particular *Camelina* accessions the results showed biased read mapping across the reference linkage groups (Figure 1, Table S2, Figure S6). In particular the *C. neglecta* accession (PI650135) aligned significantly to six chromosomes; whereas, *C. microcarpa* accessions aligned to either thirteen or 20 chromosomes. For a proportion of the *C. microcarpa* lines showing read alignment to thirteen chromosomes it was observed that the read depth was somewhat higher for six of those chromosomes, which represented the first of the three sub-genomes of the *C. sativa* hexaploid (Table S2). In light of the observed bias in read mapping, flow cytometry and chromosome counts were performed to measure the relative size of the nuclear genome content as well as to infer the ploidy level for a subset of the different *Camelina* accessions (Table 2, Figure 2, Figure S1). *Camelina sativa* (TMP23992) a well-characterized hexaploid with a genome size estimated to be 1.50 pg/2C (Martin *et al.* 2017) was used as an internal standard to measure the absolute genome size of lower ploidy *Camelina* species.

For the known diploid *C. neglecta* (2n = 12) genotype (PI650135) (previously *C. microcarpa*) the GBS data mapped to only six chromosomes thus correlated well with the expected results. This line also had the lowest genome size (0.43 pg/2C) in comparison to *C. sativa* (1.50 pg/2C). Also as expected the diploid species, *C. hispida* was found to have 2n = 14 chromosomes with a relatively similar genome size of 0.59 pg/2C as of diploid *C. neglecta*. For the *C. hispida* GBS reads, there was a significant bias in mapping with just over 57% of the reads mapped to the third subgenome of the reference *C. sativa* genome (Figure 1, Figure S6). This might indicate an affinity of *C. hispida* with the third subgenome of reference *C. sativa* (Mandáková *et al.* 2019).

**Figure 3:**
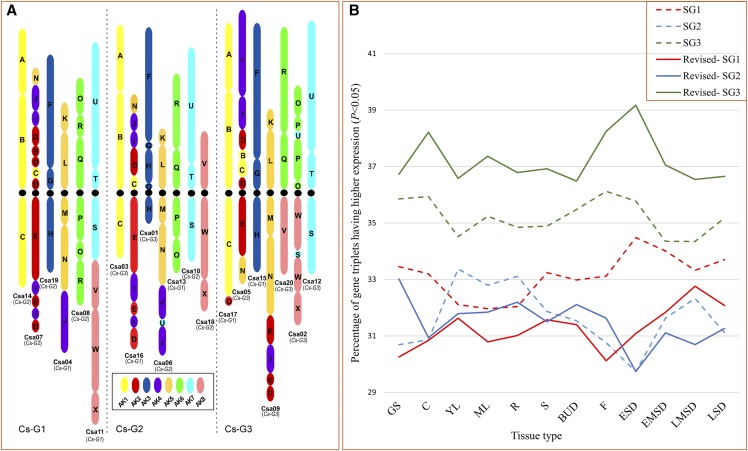
Re-defining the *Camelina sativa* subgenome composition. a) Newly defined subgenome architecture of *C. sativa*; b) Evidence of genome dominance based on refined subgenome structure and gene expression data (GS: Germinating Seed, C: Cotyledon, YL: Young Leaf, ML: Senescing Leaf, R: Root, S: Stem, BUD: Bud, F: Flower, ESD: Early Seed Development; EMSD: Early Mid Seed Development, LMSD: Late Mid Seed Development and LSD: Late Seed Development).

More interestingly, of the *C. microcarpa* lines where the GBS data aligned with 13 linkage groups from the reference genome, only one genotype (CN119243) possessed a lower genome size (0.95 pg/2C) in comparison to the hexaploids, and based on the read alignments as well as chromosome counts was inferred to be tetraploid (2n = 26) (Figure 1 and 2). Seven genotypes from *C. microcarpa* (hereafter referred to as “Type 1”) showed consistent read coverage across all chromosomes from the reference genome of *C. sativa*, while GBS data from 18 *C. microcarpa* genotypes (hereafter referred to as “Type 2”) aligned with only 13 linkage groups but with a somewhat higher read coverage in the first subgenome (Table S2). *Camelina microcarpa* (TMP24026*)*, representing the “Type 1” group, had 2n = 40 chromosomes, as expected. However, *C. microcarpa* (TMP23999), representing the “Type 2” group, had an estimated DNA content (1.49 pg/2C) similar to that of *C. sativa* yet was found to have 38-40 chromosomes, most likely 2n = 38 (Figure 2). Estimates for this latter line were slightly confounded by the large variation in size between chromosomes and are hence presented with reasonable but not 100% certainty. Sub-genome 1 of *C. sativa*, with only six chromosomes possesses a larger “fusion” chromosome (Csa-11), it would seem likely that the unidentified six chromosome sub-genome of Type 2 *C. microcarpa* has a similar “fusion” chromosome which would interfere with accurate chromosome counts; see Figure 3a.

Of the 13 chromosomes showing read alignment for the *C. microcarpa* “Type 2” group, six chromosomes were shared with the diploid species *C. neglecta* and seven with subgenome 2 of *C. sativa*, while the apparently missing chromosomes comprise subgenome 3, to which reads from the diploid *C. hispida* also align. These results suggested two different types of higher chromosome number *C. microcarpa* accessions (Type 1: 2n = 40 and Type 2: 2n = 38) with similar genome sizes; one which shares the genome organization as that of the reference *C. sativa* genome and the second which shares only two subgenomes with that of the reference. Thus, representatives of diploid, tetraploid and two different hexaploid *Camelina* “species” could be differentiated. The tetraploid *C. rumelica* (TMP24027) (Martin *et al.* 2017), previously suggested as a progenitor of *C. sativa* (Mandáková *et al.* 2019), had a higher nuclear genome content (1.26 pg/2C) than the tetraploid *C. microcarpa* (CN119243; 2n = 26). The read alignment data of *C. rumelica* mapped to all chromosomes with no observable pattern; this ambiguity with regards to its relationship to the subgenomes of *C. sativa* would not be expected if *C. rumelica* was indeed a progenitor genome (Table S2, Figure S6). Further accessions of this line would need to be tested.

### A refined subgenome structure for C. sativa

The increase in ploidy level in *Camelina* species from 2n = 12 in *C. neglecta* to 2n = 26 and 2n = 40 in *C. microcarpa* might be expected to correspond to the three subgenomes of *C. sativa* as defined in the reference genome (Kagale *et al.* 2014); however, this was not the case. The original assignment of reference pseudo-molecules to each of the subgenomes used synteny analyses to identify the most parsimonious route, minimizing genome-restructuring events, from the ancestral karyotype of the Brassicaceae to the modern day *C. sativa* genome (Kagale *et al.* 2014). However, it was recognized at the time that some linkage groups, for example Csa14 and Csa03, shared the same basic chromosome structure and their subgenome assignment was more difficult. Thus based on the GBS read alignments and the assumption that the simplest path to the hexaploid genome is through the hybridization of identified lower chromosome number species the subgenome structure has been refined. More explicitly it was assumed that *C. neglecta* is an extant relative of subgenome 1, the tetraploid *C. microcarpa* CN119243 represents the second stage in the evolutionary path and is composed of subgenome 1 and 2, and finally hexaploid *C. microcarpa* (2n = 40) is a direct ascendant of *C. sativa*, comprised of all three subgenomes; where the origin of the third subgenome is still unclear, although likely a relative of *C. hispida*. Thus the new genome organization is as follows Subgenome 1 (SG1) contains Csa14, Csa07, Csa19, Csa04, Csa08 and Csa11, which are shared with the diploid *C. neglecta* (formerly *C. microcarpa*); SG2 is composed of Csa03, Csa16, Csa01, Csa06, Csa13, Csa10 and Csa18 that along with SG1 are in common with the tetraploid *C. microcarpa* CN119243; and finally SG3 that is found in all *C. sativa* lines consists of Csa17, Csa05, Csa15, Csa09, Csa20, Csa02 and Csa12, which are also shared with *C. hispida* (Figure 1, Figure 3a). As shown in Figure 3a the majority of the re-assignments were between SG1 and SG2, with four chromosomes changing in each instance, only two chromosomes from SG3 were re-assigned. There was no suggestion of chromosomal rearrangements, although this will have to be confirmed through either genetic mapping and/or genome sequencing of the lower ploidy species. It was noted that one scaffold assigned to SG3 was found to have a high read depth when reads were aligned from *C. microcarpa* “Type 2”, which was an anomaly in the mapping pattern and could indicate a miss-assembly, which again will need to be confirmed through sequencing. The refined subgenome organization was used for all subsequent analyses.

### Population differentiation in Camelina species

Depending upon the distribution of the read alignments against the reference genome and corroborated by the chromosome counts and nuclear DNA content, only one genotype each belonged to *C. neglecta*, tetraploid *C. microcarpa*, *C. hispida* and *C. laxa*; two genotypes were classified as *C. rumelica*, and two as *C. alyssum*; seven genotypes were hexaploid *C. microcarpa* with 20 chromosomes, while, 18 genotypes belonged to *C. microcarpa* “Type 2” with putatively 19 chromosomes and a novel hexaploid structure compared to the *C. sativa* reference genome (*e.g.*, TMP23999); the remaining 160 genotypes were classified as *C. sativa* with 20 chromosomes (Table S1).

Prior to filtering, variant calling in all 193 genotypes yielded 102,744 SNPs across the *C. sativa* reference genome where a significant proportion of SNPs were from the related species (Table S3). Due to the presence of these distant relatives and the presumption of novel alleles being captured, raw SNPs were filtered for a minor allele frequency of greater than 1% among all samples and after allowing varying levels of missing data points (Figure S2), SNPs with 20% of the genotypes with missing data were selected, providing 4803 variants including indels for all the *Camelina* species studied (Figure 1). These SNPs were further filtered for indels yielding 4268 SNPs which were used to study population structure and genetic diversity in *Camelina* species.

The SNP distribution across the subgenomes reflected the genome composition of the total collection of accessions; with the first subgenome having a greater number of SNPs in comparison to the second and third; and the third subgenome having the lowest number of SNPs (Table 1). Gene diversity was found to be low for all chromosomes, similarly the PIC values were low; however, the range for these parameters was high across all chromosomes (Table 1). These results were somewhat skewed due to the genotypes from *C. microcarpa* “Type 2” and other related species which led to lower coverage in the third subgenome therefore an independent analysis was performed with the 169 genotypes with the same 20 chromosomes as that of the reference genome (Table S4). Removing the related *Camelina* species reduced the overall number of SNPs but also filtered out less polymorphic loci leading to higher average gene diversity and average PIC values for each of the chromosomes. Likewise, the analysis among the genotypes of domesticated *C. sativa* species (162 genotypes) including *C. alyssum* and *C. sativa* ssp. *pilosa* suggested an overall gene diversity of 0.181 and PIC value of 0.15 (Table S5).

**Table 1:** Genetic diversity parameters for 193 *Camelina* genotypes belonging to 8 species. The numbers in parenthesis indicate range

Subgenome	Chromosome	Total SNP	Filtered SNP	Gene Diversity	PIC
SGI	Chr14	5754	263	0.117 (0.021-0.499)	0.103 (0.020-0.375)
	Chr7	6280	235	0.130 (0.021-0.499)	0.114 (0.021-0.374)
	Chr19	5209	298	0.111 (0.021-0.500)	0.098 (0.020-0.375)
	Chr4	5462	271	0.127 (0.021-0.500)	0.111 (0.021-0.375)
	Chr8	5535	309	0.101 (0.021-0.500)	0.091 (0.020-0.375)
	Chr11	9593	550	0.120 (0.021-0.500)	0.105 (0.021-0.410)
	Subtotal	37833	1926	0.118 (0.021-0.500)	0.104 (0.020-0.410)
SGII	Chr3	3642	166	0.117 (0.021-0.498)	0.102 (0.021-0.374)
	Chr16	4333	207	0.135 (0.021-0.500)	0.118 (0.021-0.375)
	Chr1	3406	195	0.112 (0.021-0.495)	0.101 (0.020-0.372)
	Chr6	3477	153	0.146 (0.021-0.500)	0.126 (0.021-0.375)
	Chr13	3337	146	0.110 (0.021-0.499)	0.097 (0.021-0.375)
	Chr10	3614	208	0.119 (0.021-0.500)	0.104 (0.021-0.375)
	Chr18	2740	167	0.111 (0.021-0.495)	0.099 (0.021-0.373)
	Subtotal	24549	1242	0.122 (0.021-0.498)	0.107 (0.021-0.374)
SGIII	Chr17	5200	139	0.102 (0.021-0.397)	0.094 (0.021-0.318)
	Chr5	4993	156	0.137 (0.021-0.500)	0.120 (0.021-0.375)
	Chr15	4726	152	0.082 (0.021-0.406)	0.075 (0.021-0.324)
	Chr9	6603	186	0.084 (0.022-0.499)	0.076 (0.022-0.374)
	Chr20	5031	105	0.089 (0.021-0.494)	0.079 (0.021-0.372)
	Chr2	4451	122	0.099 (0.021-0.498)	0.089 (0.021-0.374)
	Chr12	6450	188	0.106 (0.021-0.494)	0.093 (0.021-0.372)
	Subtotal	37454	1048	0.100 (0.021-0.470)	0.089 (0.021-0.359)
Scaffolds		2908	52		
**Total SNPs**		**102744**	**4268**	**0.114 (0.020-0.500)**	**0.101 (0.000-0.410)**

**Table 2:** Genome size estimation of different *Camelina* species using flow cytometry

Species	Accession	2C DNA (pg)	Ploidy
*C. neglecta*	PI650135	0.43 ± 0.01	2x
*C. hispida*	PI650133	0.59 ± 0.02	2x
*C. microcarpa* “4x”	CN119243	0.95 ± 0.02	4x
*C. rumelica*	TMP24027	1.26 ± 0.02	4x
*C. microcarpa* “Type 2”	TMP23999	1.49 ± 0.03	6x
*C. sativa*	TMP23992	1.50 ± 0.03	6x

**Figure 4:**
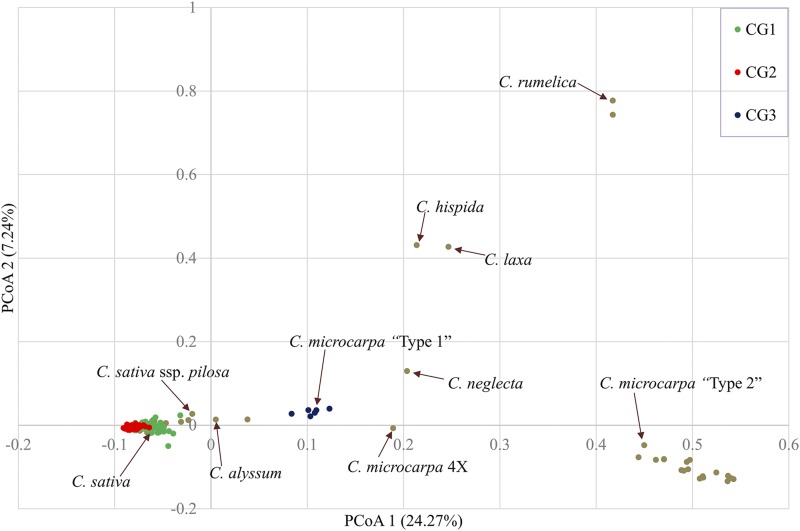
Principle coordinate analysis of 193 *Camelina* genotypes based on 4268 SNPs. The different colors represent three subpopulations defined by the STRUCTURE analysis.

Principle coordinate analysis (PCoA) differentiated the related species from the *C. sativa* population including *C. alyssum* and *C. sativa* ssp. *pilosa* (Figure 4). The first coordinate explains 24.27% of the variation, which differentiated *C. sativa* from other *Camelina* relatives; the second coordinate explains 7.24% of variation, which differentiated more distant relatives such as *C. rumelica*, *C. laxa* and *C. hispida* from *C. sativa* and *C. microcarpa*. The PCoA result suggested that *C. alyssum* followed by *C. microcarpa* “Type 1” genotypes were quite similar to domesticated *C. sativa*, while *C. microcarpa* “Type 2”, *C. hispida*, *C. laxa* and *C. rumelica* species were clearly divergent. This analysis mainly differentiated between species; however, separate analysis of *Camelina* species with 20 chromosomes was used to differentiate among *C. sativa* genotypes, and to suggest some sub-population structure (Figure S3).

**Figure 5:**
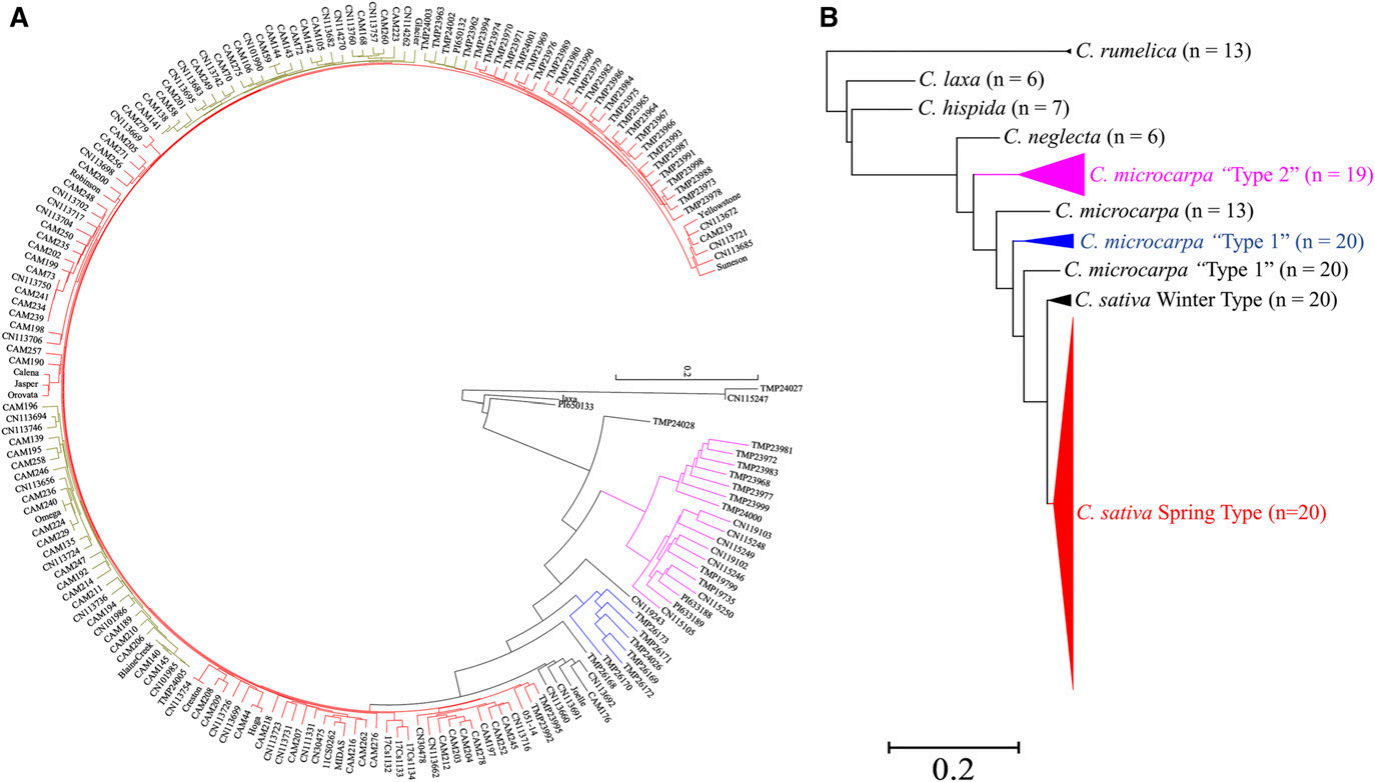
Genetic relationship among *Camelina* accessions as determined by NJ tree construction based on 4268 SNPs. a) Relationship among 193 *Camelina accessions*; b) Summary of the relationship among different species of *Camelina* (number in parenthesis indicate number of chromosomes in a haploid set).

The results from the PCoA were mirrored in the generation of a Neighbor Joining (NJ) tree showing the phylogenetic relationships among the 193 *Camelina* genotypes (Figure 5). All the domesticated *Camelina* genotypes were closely related to each other, forming a separate large cluster. The NJ tree showed that the related species, which all share a vernalisation requirement, were clustered next to a number of *Camelina* lines which were winter types, including *C. alyssum* (CAM176), *C. sativa* ssp. *pilosa* (CN113692) and the line Joelle (North Dakota State University) (Figure 5). Tetraploid *C. microcarpa* CN119243 formed a separate cluster and was basal to the *C. sativa* sub-populations, the diploid *C. neglecta* (PI650135) was basal to all higher chromosome number accessions. One *C. microcarpa* genotype (TMP26168) had a very similar genomic organization as the reference genome; however, was categorized as *C. microcarpa* “Type 1” and formed a separate single cluster. *Camelina microcarpa* “Type 2” species formed their own separate cluster, but showed further sub-population structure, separating into two groups with 11 and 7 genotypes, respectively. Two genotypes belonging to *C. rumelica* formed a separate cluster along with *C. laxa* and *C. hispida* and suggesting these had diverged sometime earlier from the progenitors of domesticated *Camelina* species.

**Figure 6:**
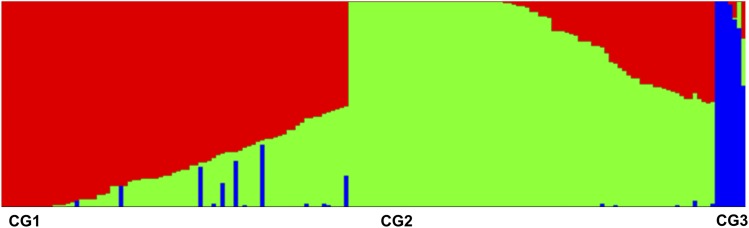
Population structure of *Camelina* species. CG1 (Red) and CG2 (Green) represent *C. sativa* genotypes, and CG3 (Blue) represents *C. microcarpa* “Type 1”.

The PCoA and NJ suggested some sub-structure among the domesticated *C. sativa* accessions, which was further assessed using the Bayesian clustering approach of STRUCTURE (Pritchard *et al.* 2000). This analysis was performed with the hexaploid *Camelina* accessions with 20 chromosomes only (n = 169) and suggested two populations confirming the separation of *C. microcarpa* “Type 1” accessions from *C. sativa*. The peak of delta K also suggested further population differentiation at K = 3, which identified two sub-populations among the *C. sativa* accessions. Assuming this three population structure and, based on a Q value cut-off of 70%, 124 genotypes were clustered into three subpopulations with 45 genotypes found to be an admixture of these subpopulations (Table S6, Figure S4). As shown in Figure 6, 162 *Camelina* genotypes were found in two sub-populations CG1 (red), CG2 (green) and *C. microcarpa* “Type 1” formed subpopulation CG3 (blue). The genotypes belonging to CG1 and CG2 were spring type whereas the genotypes belonging to CG3 were winter type. One genotype (TMP26168) belonging to *C. microcarpa* “Type 1” was found to be an admixture of CG3, CG2 and CG1, which confirmed its unique status, noted in the NJ tree analyses. The winter type *C. alyssum* (CAM176) was also an admixture of CG1, CG2 and CG3, with a higher contribution from subpopulation CG1. Other winter types such as *C. sativa* ssp. *pilosa* (CN113692) and *C. sativa* (Joelle) were grouped with CG1. All the winter type *Camelina* lines were found to have a contribution of alleles from subpopulation CG3, representing *C. microcarpa* “Type 1” (Table S6).

Pairwise F_ST_ values were calculated among the three subpopulations (124 genotypes), excluding the lines showing admixture. The results suggested that spring type *Camelina* species of subpopulations CG1 and CG2 were closely related with an F_ST_ of 0.065. F_ST_ values between the two spring *Camelina* sub-populations and *C. microcarpa* “Type 1” indicated greater differentiation between the species, with values of 0.302 and 0.349, respectively (Table 3). However, a separate analysis of pairwise F_ST_ with all the genotypes irrespective of admixture suggested a lower F_ST_ value (0.263) (Table S7d). For all the subpopulation the third subgenome showed higher differentiation among subpopulations in comparison to the other subgenomes (Table S7). The F_ST_ analysis between *C. sativa* and *C. microcarpa* “Type 1” also suggested strong selection for alleles in *C. sativa* on chromosome Csa06 in a relatively small region (6Mb to 9 Mb region) (Figure 1).

**Table 3:** Pairwise F_ST_ among three subpopulations of *Camelina* species. CG1 (58 genotypes) and CG2 (60 genotypes) represent *C. sativa* genotypes and CG3 (6 genotypes) represents *C. microcarpa* “Type 1” accessions

	CG1	CG2	CG3
**CG1**	0.000		
**CG2**	0.065	0.000	
**CG3**	0.302	0.349	0.000

### Related Camelina species as a reservoir of minor alleles

**Figure 7:**
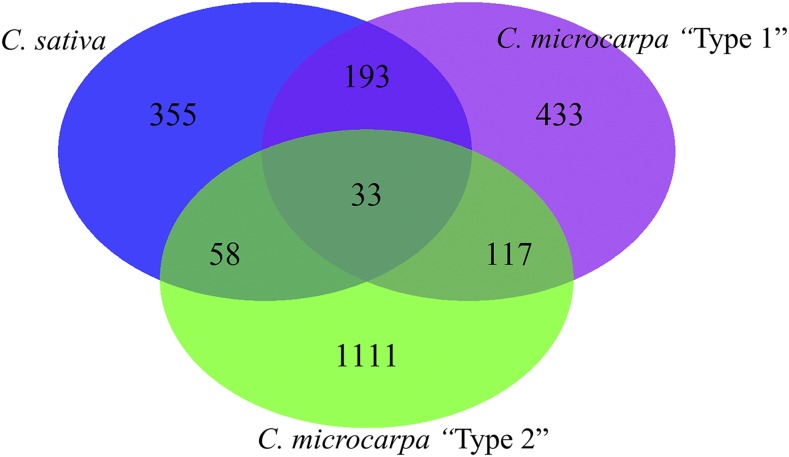
Venn diagram showing distribution of minor alleles in different species of *Camelina*.

Although, this study included a number of species, approximately 96% of the total samples were either classified as *C. sativa*, *C. microcarpa* “Type 1” or *C. microcarpa* “Type 2”. Among the 4268 filtered SNPs, the number of minor alleles (less than 5% homozygous) were identified for each of the three species, to assess their potential as a source of novel alleles. Such minor alleles were found for 2300 SNPs; only 33 were shared by all three species (Figure 7). Of the minor alleles, 1111 were unique to *C. microcarpa* “Type 2”, 433 were unique to *C. microcarpa* “Type 1” and 355 were unique to *C. sativa* species. The distribution of minor alleles along the subgenomes suggested the first subgenome of both *C. sativa* and *C. microcarpa* “Type 2” contained the highest number of minor alleles, while the third subgenome for *C. microcarpa* “Type 1” contained more minor alleles (Table S8).

Minor alleles not present in the domesticated *C. sativa* were explored to identify mutations that may have helped to shape the existing *C. sativa* accessions through selection for changes to particular genes. Of all the SNPs with minor alleles 536 were within the genic region of 355 genes. Of these, 275 genes had orthologs in *Arabidopsis thaliana* (Table S8a), although there was no apparent bias for particular functional category, three genes were found to have an influence on flowering time and photoperiod response and could be interesting candidates for manipulating phenology (Table S8b).

## Discussion

The current study exploited GBS data and the reference genome of *C. sativa* to characterize variation among *Camelina* species, which not only identified a potentially novel *Camelina* species but also suggested refinements to the underlying subgenome structure of *C. sativa*. The hexaploid structure of *C. sativa* was clear from the genome assembly of Kagale *et al.* (2014); however, the differentiation of the three subgenomes was complicated by the high degree of synteny between particular chromosomes. Phylogenetic analyses of a set of unanchored genome scaffolds of *C. neglecta* (PI650135) (Toro 2017) also suggested changes to the first subgenome of *C. sativa* genome, which concurred with the GBS data presented in this study. By alignment of GBS data from the diploid *C. neglecta* (2n = 12), a presumed tetraploid (*C. microcarpa*; 2n = 26) and multiple hexaploids (2n = 40) a step-wise hybridization path to the current *C. sativa* genome was suggested, implicating the diploid and tetraploid line as potential progenitor species of *C. sativa*. The third subgenome shares significant homology to *C. hispida*, implying this may represent an extant progenitor of the final subgenome, which is in agreement with the recent work of Mandáková *et al.* (2019).

After redefining the subgenome composition of *C. sativa*, there was a slight change in distribution of gene coverage, with a higher number of genes now present on the third subgenome (33.7% compared to 32.7% of total annotated genes) and a slight decrease in the number of genes for the second subgenome (30.2% compared to 31.1% of total genes) (Table S9). Although there was no change in number of genes retained in triplicate, in light of the re-definition of the karyotype, subgenome dominance was re-analyzed based on the previously published gene expression data from Kagale *et al.* (2016). Depending on the tissue type between 9,188 (late seed development) and 12,688 (root) triplicated orthologous gene sets were analyzed for evidence of genome dominance in *C. sativa* (Table S10). As found in Kagale *et al.* (2016) the results suggest dominance of the third subgenome over the other two; however, the impact was far more pronounced (Figure 3b). For all tissue types, the third subgenome had a greater number of genes with higher expression in comparison to both the first and second subgenome, deviating from a hypothetical 1:1:1 ratio of number of genes significantly expressing higher in any one subgenome (*χ*^2^ test, *P-value * < 0.05). There were some tissue specific patterns observed with regards to SG1 and SG2: the second subgenome was found to dominate the first subgenome until flowering, after which the first subgenome dominated the second. However, the ratio of the total number of expressed genes for the third subgenome with either first or second subgenome was not particularly high (∼1.11-1.27), suggesting limited gene silencing, and might reflect the young neopolyploid status of *Camelina* as suggested by Kagale *et al.* (Kagale *et al.* 2014). The marked dominance of the third subgenome, or by inference the genome added last in the stepwise evolution of *C. sativa*, is in concordance with evidence from other polyploid species with similar evolutionary trajectories (Ramírez-González *et al.* 2018; Edger *et al.* 2019; Mandáková *et al.* 2019).

The chromosome numbers for *C. neglecta*, *C. hispida*, *C. sativa and C. microcarpa* “Type 1” were consistent with previous reports (Martin *et al.* 2017; Brock *et al.* 2018). However, *C. microcarpa* “Type 2” was suggested to have n = 19 chromosomes, noticeably the sequences from this genome mapped to only two of the *C. sativa* subgenomes, suggesting a hexaploid derived from progenitors with 6, 7 and 6 chromosomes. The available tetraploid (n = 13) which could be a progenitor of both “Type 1” and “Type 2” *C. microcarpa* suggests two different routes to the formation of the higher ploidy hexaploid genomes in the *Camelina* genus. The mapping of *C. hispida* (n = 7) to the third subgenome of *C. sativa* (Figure 1), also indicated by the results of Mandáková *et al.* (2019) could suggest hybridization of the tetraploid with *C. hispida* in the formation of modern hexaploid *C. sativa*. As yet, the origin of the third subgenome for *C. microcarpa* “Type 2” remains elusive, although it shares some homology with subgenome 1, suggesting it could be a relative of *C. neglecta*. The current study did not find clear association of the tetraploid *C. rumelica* with specific subgenomes of the reference *C. sativa*, suggesting that greater genetic distance and possibly chromosomal rearrangement separate the two species (Čalasan *et al.* 2019).

The genetic characterization of the accessions confirmed the low level of differentiation among *C. sativa* lines (Vollmann *et al.* 2005; Singh *et al.* 2015; Luo *et al.* 2019; Gehringer *et al.* 2006), yet there was some indication of sub-structure within the *C. sativa* population. A significant number of recently collected accessions, which originated from the Russian/Ukraine border populated CG1 and could provide a source of some limited variation in *C. sativa* breeding, but the related hexaploid species offer the potential of much more diversity. It appears that some of this variation may have begun to be captured, in particular with the generation of *C. sativa* types with a vernalisation requirement. Similarly, it was noted that one apparent *C. microcarpa* “Type 1” line showed evidence of shared alleles across the three defined sub-populations, including those seemingly specific to *C. sativa*. The evolutionary history of *Camelina* hexaploids may have played a role in limiting variation with a smaller number of SNPs found in the second subgenome, which may reflect a small number of hybridization events from which this subgenome was derived. Although *C. sativa* and *C. microcarpa* both evolved through polyploidy, *C. microcarpa* “Type 1” has maintained a greater collection of minor alleles, implicating the influence of selection on a crop which has been subjected to less intensive breeding than most, or again could result from a polyploidization bottleneck. The frequency of minor alleles was higher in the first subgenome of domesticated *C. sativa* in comparison to *C. microcarpa* “Type 1” (Table S8) and might indicate further differentiation of *C. sativa* subpopulations or relate to age of divergence of the subgenomes. The study of minor allele frequencies has been used to understand domestication and potential bottlenecks created during the process, enabling the identification of genes under selection that may underlie QTL controlling traits of interest (Ross-Ibarra *et al.* 2007). The current study identified a number of genes carrying minor alleles in the wild relative that may represent genes under selection in the crop, further comprehensive sequence analyses and trait association will determine the value of such variation.
